# Bidirectional recurrent learning of inverse dynamic models for robots with elastic joints: a real-time real-world implementation

**DOI:** 10.3389/fnbot.2023.1166911

**Published:** 2023-06-16

**Authors:** Brayan Valencia-Vidal, Eduardo Ros, Ignacio Abadía, Niceto R. Luque

**Affiliations:** ^1^Department of Computer Engineering, Automation and Robotics, Research Centre for Information and Communication Technologies, University of Granada, Granada, Spain; ^2^Research Group Osiris & Bioaxis, Faculty of Engineering, El Bosque University, Bogotá, Colombia

**Keywords:** robot dynamic modeling, gated recurrent units, bidirectional recurrent neural networks, compliant robots, torque control

## Abstract

Collaborative robots, or cobots, are designed to work alongside humans and to alleviate their physical burdens, such as lifting heavy objects or performing tedious tasks. Ensuring the safety of human–robot interaction (HRI) is paramount for effective collaboration. To achieve this, it is essential to have a reliable dynamic model of the cobot that enables the implementation of torque control strategies. These strategies aim to achieve accurate motion while minimizing the amount of torque exerted by the robot. However, modeling the complex non-linear dynamics of cobots with elastic actuators poses a challenge for traditional analytical modeling techniques. Instead, cobot dynamic modeling needs to be learned through data-driven approaches, rather than analytical equation-driven modeling. In this study, we propose and evaluate three machine learning (ML) approaches based on bidirectional recurrent neural networks (BRNNs) for learning the inverse dynamic model of a cobot equipped with elastic actuators. We also provide our ML approaches with a representative training dataset of the cobot's joint positions, velocities, and corresponding torque values. The first ML approach uses a non-parametric configuration, while the other two implement semi-parametric configurations. All three ML approaches outperform the rigid-bodied dynamic model provided by the cobot's manufacturer in terms of torque precision while maintaining their generalization capabilities and real-time operation due to the optimized sample dataset size and network dimensions. Despite the similarity in torque estimation of these three configurations, the non-parametric configuration was specifically designed for worst-case scenarios where the robot dynamics are completely unknown. Finally, we validate the applicability of our ML approaches by integrating the worst-case non-parametric configuration as a controller within a feedforward loop. We verify the accuracy of the learned inverse dynamic model by comparing it to the actual cobot performance. Our non-parametric architecture outperforms the robot's default factory position controller in terms of accuracy.

## 1. Introduction

The presence of robots among us is becoming increasingly common as technology provides them with new capabilities needed for performing diverse tasks in unstructured, scenarios, i.e., healthcare centers, homes, etc. In fact, the International Federation of Robotics (IFR) reported ~3 million robots operating in 2020, a 10% increase compared to the previous year (IFR, 2021). This ubiquitous presence entails an increasing human–robot interaction (HRI) in a collaborative manner. Interacting with a human can be regarded as an unstructured task because it is ambiguous, and it is not based on rigid rules. Rather, HRI tasks require general policies incorporating high rates of exception and complexity, in which adaptability and flexibility are mandatory (Bicchi et al., 2008). It is precisely the need for this HRI to be safe that motivates the emergence and design criteria for collaborative robots, also called cobots (Giuliani et al., 2010).

The latest cobot generation integrates elastic actuators that offer passive compliance and minimizes execution forces to comply with safe HRI while performing unstructured tasks (Giuliani et al., 2010). These elastic joints reduce the risk of damage due to the dampening effect over the contact forces between the cobot and a stiff environment, i.e., cobot–cobot, cobot–operator, or cobot–environment contact. However, the integration of the elastic components entails a trade-off: an increase in the complexity of the cobot dynamics and modeling, which, in turn, presents challenges in control (Lee et al., 2017). The dynamic model of any robot relates joint torque values to their resultant joint motion, which implies that position and velocities could be estimated from the applied torque commands (direct dynamic model) and vice versa (inverse dynamic model). The classical methods for identifying a robot dynamic model are based on either the Lagrange equations (analytical dynamic model) or the Newton–Euler equations (numerical dynamic model; Swevers et al., 2007).

Although flexible joint robot dynamic identification is a viable option for modeling compliant cobots, this method has limitations in accurately capturing the model's parameters and the non-linear and time-varying dynamics (Pratt and Williamson, 1995). Simple analytical dynamic models often do not consider joint stiffness and elasticity or, if they do, they approximate linear behavior with coefficients that have a high degree of uncertainty (Kwon and Book, 1994; Ata et al., 1996; Calanca et al., 2011; Lee et al., 2017). Additionally, other non-linear effects such as backlash and frictional torque are often overlooked (Madsen et al., 2020). The dynamics of these effects pose a challenge to the accuracy of the model as they are affected by environmental conditions and the level of maintenance of the robot. Therefore, data-driven dynamic modeling using Machine Learning (ML) approaches, specifically artificial neural networks (ANNs), has gained popularity. ANNs are capable of capturing complex and non-linear dynamics, making them well-suited for this purpose (Xu et al., 2014; Li and Li, 2021; Liu et al., 2021). This is particularly important in tasks that involve close human-robot interaction, where ensuring the safety of human operators is critical. It is worth noting, however, that modeling flexible joints in robot dynamic identification may still be useful in certain scenarios. Nevertheless, the limitations of this approach have motivated the exploration of alternative methods such as data-driven dynamic modeling using ML techniques.

Several research studies (Graves et al., 2005; Rueckert et al., 2017; Liu et al., 2019) have consistently reported better performance of Recurrent Neural Networks (RNNs), specifically bidirectional RNNs (BRNNs), over non-recurrent ANNs in time-series problems (of a moderate number of inputs), such as the one we are dealing with. RNNs are extensively used to model different dynamic systems (Jin et al., 2022). Particularly, in robotics, unidirectional RNNs have been trained to learn the inverse or direct dynamics of rigid robots (Mukhopadhyay et al., 2019; Wang et al., 2020). Hybrid solutions combining an analytical description of the rigid robot dynamics with a data-driven deep learning (DL) model (semi-parametric model) has also been proposed (Liu et al., 2020; Çallar and Böttger, 2023). Conversely, in cobotics, unidirectional and BRNNs have been trained to learn cobot models only relating desired joint positions to actual joint positions, without taking into account the actual torque values that are required to achieve these positions (Chen and Wen, 2019). This limitation can restrict the application of these models to torque control, where knowledge of the actual torque values is crucial. Note that the majority of these studies were focused on the modeling of rigid robots and never on the application of ML to robots with elastic joints that are inherently safer, but difficult to be commanded in torque accurately due to their complex dynamics, i.e., passively compliant cobots. Importantly, these studies do not account for the future states of the joints of the robot, which provide relevant information for appropriate learning of an inverse dynamics model (Jordan and Rumelhart, 1992). Therefore, there is a need for research on (ML)-based inverse dynamic models that can account for the complex dynamics of passively compliant cobots and physical force interactions, ultimately facilitating model-free torque control. We propose and evaluate three machine learning algorithm configurations based on recurrent neural networks (RNNs).

Three approaches can be found in dynamic systems modeling depending on whether the physical parameters of the system are incorporated or not, i.e., parametric, non-parametric, and semi-parametric models. We proposed a configuration that builds a non-parametric model (NID) and two configurations that build semi-parametric models (SID and ESID) incorporating the robot's rigid body parametric model (RBD). Specifically, we wanted to determine whether combining the RBD model with learning models (BRNN) could provide greater robustness in prediction and generalization than when the RBD model was not taken into account. We also wanted to investigate whether the way in which information from the RBD model was incorporated into the semi-parametric models influenced their performance. In the SID configuration, the output of the RBD model is an input to the BRNN, whereas, in the ESID configuration, the BRNN is trained to estimate the torque corresponding to the dynamics not modeled in RBD so that the outputs of the BRNN and RBD can be combined.

Considering all the implications above, we can enumerate the main contributions of this work as follows:

- First, this study proposes an efficient dynamic data robot acquisition method. Using statistical analysis over the dataset, we identify the subset of trajectories that provided the highest dynamic cobot information, i.e., good data vs. extensive big data paradigm. The resulting training dataset is made publicly available for replication and comparison purposes.
- Second, we introduce an ML approach to the inverse dynamic modeling of cobots based on three different BRNN configurations: a non-parametric BRNN, i.e., data-driven model and two semi-parametric BRNNs, i.e., data-driven model + analytical rigid-dynamic model. We compare these data-driven models to the analytical rigid-dynamic model provided by the manufacturer and find that the BRNN-based models systematically outperform the analytical model.
- Finally, we demonstrate that implementing a BRNN-based controller that has learned the inverse dynamics of the robot can improve the performance of a cobot, even under persistent collisions or lack of active feedback. Our approach carries high accuracy while maintaining the cobot's compliance. This was illustrated as a proof-of-concept within the Supplementary video.

## 2. Materials and methods

### 2.1. Passive compliant robot

The Baxter robot^Ⓡ^ was used as our robotic demonstrator. Baxter is a collaborative and compliant robot (cobot) consisting of two arms with seven degrees of freedom (DoF) each, equipped with serial elastic actuators (SEAs) (Fitzgerald, 2013). Unlike rigid actuators, these SEAs incorporate a spring between the motor gear and the actuator end (Pratt and Williamson, 1995), which allows absorbing, to some extent, contact forces, that is, providing passive compliance. In addition to the SEAs, Baxter also holds a passive spring at the S1 joint (see Section 3), which further increases the dynamic complexity of the robot arm.

### 2.2. Cobot dynamic modeling and the parametric issue

Any robot dynamic model describes the relationship between the torque values applied on the robot joints and the resulting motion. This relationship is mathematically expressed using the analytic Lagrange formulation as follows:


(1) $$\begin{array}{l} \tau =M\left(q\right)\ddot{q}+H\left(q,\dot{q}\right)+G\left(q\right)+\xi \left(q,\dot{q},\ddot{q}\right), \end{array}$$

where *q*, $\dot{q}$, and $\ddot{q}$, are joint positions, velocities, and accelerations, respectively. τ stands for the vector containing the joint torque values. *M*(*q*)∈*R*^*n*x*n*^ defines the robot inertia matrix. The $H\left(q,\dot{q}\right)\in R^{n}$ computes the inertia and Coriolis effects, whereas *G*(*q*)∈*R*^*n*^ computes the gravitational effects onto the robot. Finally, $\xi \left(q,\dot{q},\ddot{q}\right)\in R^{n}$ stands for the torque/force effects of those robot elements that were not considered elsewhere in the dynamic model, i.e., viscous friction, or the non-linear effects of the SEA springs.

Most rigid robots, i.e., equipped with high ratio gearboxes, conveniently assume ξ = 0 in dynamic modeling since $M\left(q\right)\ddot{q}$ and $H\left(q,\dot{q}\right)$ torque contributions to the final τ are significantly larger than $\xi \left(q,\dot{q},\ddot{q}\right)$. In these cases, the torques governed by rigid body dynamics (τ_RBD_) can be defined as follows:


(2) $$\begin{array}{l} \tau_{\text{RBD}}=M\left(q\right)\ddot{q}+H\left(q,\dot{q}\right)+G\left(q\right). \end{array}$$

However, this is no longer the case for non-rigid robots (cobots), such as Baxter. Modeling $\xi \left(q,\dot{q},\ddot{q}\right)$ becomes key in associating accurately the applied cobot torque values and the subsequent motion. ξ related cobot parameter demands for accurate identification methods, which are usually mathematically intractable (Lee et al., 2017). Mathematically intractability motivates the employment of non-parametric methods for cobot dynamic modeling (Polydoros et al., 2015). Consequently, modeling an elastic robot is a challenging task that requires not only understanding or learning the dynamics of the rigid components (Equation 2) but also the additional complex dynamics inherent in its elastic joints [$\xi \left(q,\dot{q},\ddot{q}\right)$]. Accurately capturing these additional dynamics requires sophisticated techniques and algorithms, as they involve frictional and elastic behavior that cannot be captured just by rigid body dynamics. Dynamic modeling that performs well for rigid robots may not be suitable for elastic ones due to the increased complexity involved in modeling their additional complex dynamics. Therefore, modeling elastic robots requires careful consideration of their specific requirements and characteristics (Madsen et al., 2020).

Note that the Baxter rigid analytical dynamic model was used for comparative purposes and the proposed semi-parametric model. We use the Unified Robot Description Format (URDF) file provided by the manufacturer of the Baxter robot. This file contains physical parameters of the robot links such as masses, inertia tensors, and relative centers of masses. *Pybullet* library (Coumans and Bai, 2016-2021) was used to build a model of the Baxter robot with these parameters. Pybullet uses the method of Newton–Euler formulation proposed by Luh et al. (1980) for the calculation of the inverse dynamics.

### 2.3. Cobot dynamics learning: the recurrent neural network

Inverse dynamic modeling estimates those torque values needed to generate a certain cobot movement. This problem is identified in ML terms as a regression problem (Nguyen-Tuong and Peters, 2008). To solve this regression problem, i.e., estimating the relationship between a dependent variable (torque) and independent variables (joint positions, velocities, and accelerations), we used an RNN. Note that Equation (1) describes the relationship between the robot's joint torque values and its kinematic state variables [$x=\left(q,\dot{q},\ddot{q}\right)$]. The recursive Newton–Euler formulation is often used to calculate the torque values based on the robot's state at a specific instant (Luh et al., 1980). To determine the torque sequence [*Y* = (*y*_1_, ..., *y*_*T*_)] along a given trajectory, a possible approach is to first discretize the trajectory, then determine the kinematic state at each discrete instant [*X* = (*x*_1_, ..., *x*_*T*_)], and finally solve Equation (1) for each kinematic state. This approach enables us to treat the inverse dynamic problem as a time-series regression problem. By predicting the output Y based on the input sequence X [*Y* = *F*(*X*), where F is a non-linear function], we can determine the torque sequence along a given trajectory (Rueckert et al., 2017). Interestingly, recent research has shown a relationship between the state space of a model and the hidden states of RNNs (Schüssler et al., 2019; Ljung et al., 2020; Uribarri and Mindlin, 2022), leading to investigations into the potential of RNNs to predict time series of complex non-linear dynamical systems in robotics (Mohajerin and Waslander, 2019; Çallar and Böttger, 2023).

The neural network was only fed with position and velocity values as inputs, whereas the acceleration values were inferred from those position and velocity data. When velocity sensors are available, incorporating this information directly into the network is generally preferable to estimating it from joint positions. However, in cases where velocity sensors are not available or are unreliable, estimating velocity from position data can be an alternative approach, although it may introduce errors due to noise, drift over time or, more importantly, the discretization error or truncation error (Brown et al., 1992). Regarding acceleration, our robot's sensors do not provide for acceleration. Therefore, we chose to infer acceleration internally through the RNN. Specifically, the RNN can use the time-serial information from position and velocity data to estimate acceleration. While this approach may also introduce some errors, it has been shown to work well in previous studies (Liu et al., 2019).

Recurrent neural networks are traditionally implemented using cell solutions, the most notable being the Long Short Term Memory (LSTM) cell and the Gated Recurrent Unit (GRU) cell (Chung et al., 2014). LSTM cell consists of two recurrent inputs called forged gate (*c*_*t*−1_) and the input gate (*h*_*t*−1_), and the output *h*_*t*_ is defined as follows:


(3) $$\begin{array}{l} i_{t}=\sigma \left(W_{i}x_{t}+U_{i}h_{t-1}+b_{i}\right),\\ f_{t}=\sigma \left(W_{f}x_{t}+U_{f}h_{t-1}+b_{f}\right),\\ r_{t}=\sigma \left(W_{r}x_{t}+U_{r}h_{t-1}+b_{r}\right),\\ o_{t}=\sigma \left(W_{o}x_{t}+U_{o}h_{t-1}+b_{o}\right),\\ {\tilde{c}}_{t}=tanh\left(W_{\tilde{c}}x_{t}+U_{\tilde{c}}h_{t-1}+b_{\tilde{c}}\right),\\ c_{t}=\sigma \left(f_{t}*c_{t-1}+i_{t}*{\tilde{c}}_{t}\right),and\\ h_{t}=o_{t}*tanh\left(c_{t}\right). \end{array}$$

Conversely, GRU is defined by one only gate, the update gate, which reduces mathematical complexity and, therefore, computational cost (Yang et al., 2020). The equations governing the GRU behavior are as follows:


(4) $$\begin{array}{l} z_{t}=\sigma \left(W_{z}x_{t}+U_{z}h_{t-1}+b_{z}\right), \end{array}$$

(5) $$\begin{array}{l} r_{t}=\sigma \left(W_{r}x_{t}+U_{r}h_{t-1}+b_{r}\right), \end{array}$$

(6) $$\begin{array}{l} {\tilde{h}}_{t}=tanh\left(W_{\tilde{h}}x_{t}+U_{\tilde{h}}\left(h_{t-1}*r_{t}\right)+b_{\tilde{h}}\right),and \end{array}$$

(7) $$\begin{array}{l} h_{t}=\left(1-z_{t}\right)*h_{t-1}+z_{t}*{\tilde{h}}_{t}. \end{array}$$

The operator * stands for the Handamard (i.e., element-wise) product of two vectors. σ(·) and *tanh*(·) represent the sigmoid and the hyperbolic tangent activation functions, respectively. *W*_*z*_, *W*_*r*_, and $W_{\tilde{h}}$ are the weight matrices of the input (*x*_*t*_), whereas *U*_*z*_, *U*_*r*_, and $U_{\tilde{h}}$ are the weight matrices of the recurrent input gate (*h*_*t*−1_) within the cell. *b*_*z*_, *b*_*r*_, and $b_{\tilde{h}}$ refer to the bias vectors. *r* and *z* are the reset and update gates, respectively. ${\tilde{h}}_{t}$ is the candidate output, whereas *h*_*t*_ is the output of the cell unit at time *t*. *h*_*t*_ also stores the information of the previous state. Note that we selected the GRU cells for the RNN due to their lower computing cost (Yang et al., 2020; see Section 3.3).

**Figure 1:**
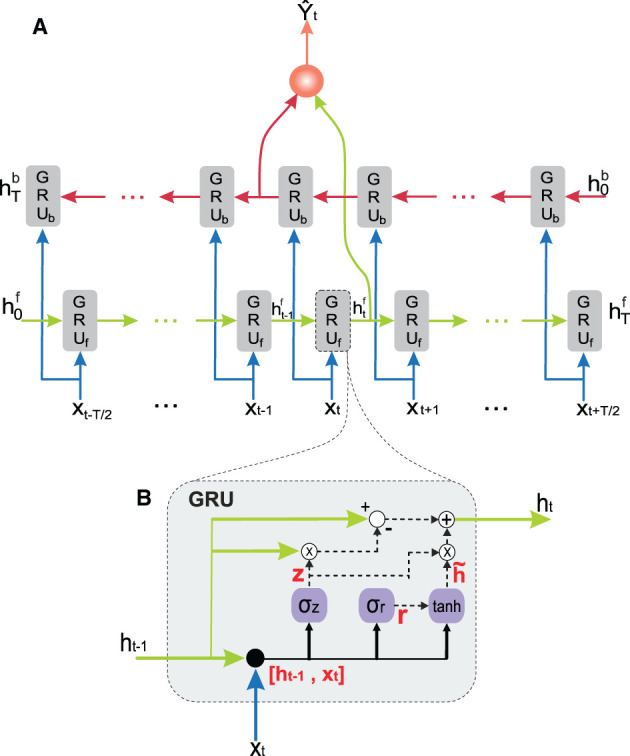
Structure of the proposed neural network. **(A)** Bidirectional recurrent neural network (BRNN) consisting of a backward (red) and a forward (green) recurrent neural network (RNN). **(B)** RNN cell solution; the gated recurrent unit (GRU).

We also incorporated an ANN readout layer to each GRU cell since cell and hidden states are the same (Figure 1). We assembled these GRU cells following a BRNN model (Graves et al., 2005). In our BRNN implementation, we used two independent RNNs to process the input sequence in both forward and backward directions. This approach allowed us to take into account both past and future contexts when predicting the output values. By using two separate RNNs, we could achieve improved performance compared to a unidirectional RNN. One RNN handled positive time direction (GRU_*f*_), i.e., information from the past toward the current state, whereas the other RNN handled negative time direction (GRU_*b*_), i.e., information from the future toward the current state (Schuster and Paliwal, 1997). We fed the model with target future states of the robot, i.e., following desired joint position and velocity values.

**Figure 2:**
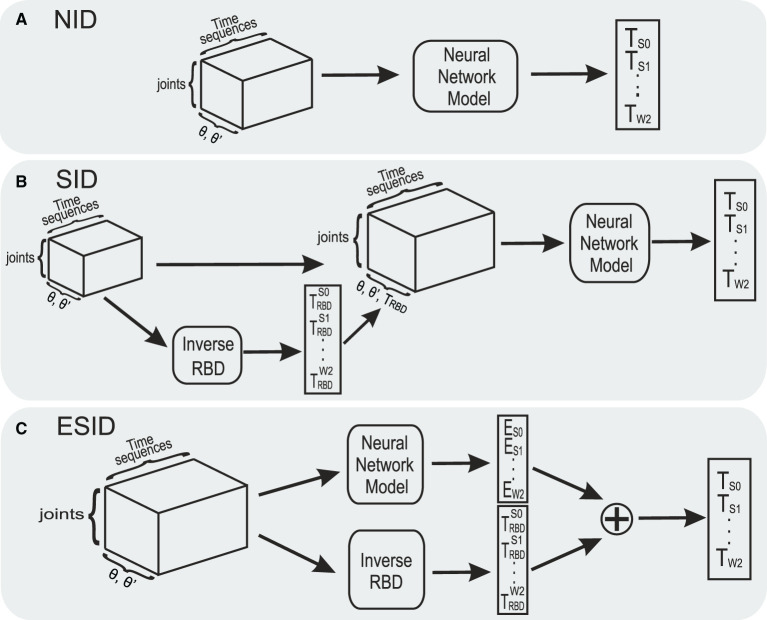
Three proposed inverse dynamic modeling configurations using a BRNN network. **(A)** Non-parametric configuration (NID). **(B)** Semi-parametric configuration (SID) receiving joint positions, velocities, and estimated torques from the inverse RBD model. **(C)** Error compensator inverse dynamic (ESID) configuration compensates for the dynamics that are not modeled by the inverse RBD model.

This BRNN model was later incorporated into three different configurations (Figure 2). (i) A non-parametric inverse dynamic configuration (NID) in which the BRNN received joint positions and velocities (seven positions and seven velocity values) as inputs, and it predicted the torque values for each joint. (ii) A semi-parametric inverse dynamic configuration (SID) in which the BRNN received joint positions, velocities, and the torque estimated by the rigid body dynamic (RBD) model (described by Equation 2) as inputs, and it predicted the torque values for each joint. (iii) An error compensator semi-parametric inverse dynamic configuration (ESID) in which the BRNN received joint positions and velocities as inputs and whose outputs [$\xi_{\text{ESID}}\left(q,\dot{q}\right)$] were added to the rigid-body dynamic model to calculate the total torque values of each joint (τ_total_), as defined in


(8) $$\begin{array}{l} \tau_{\text{total}}=\tau_{\text{RBD}}+\xi_{\text{ESID}}\left(q,\dot{q}\right). \end{array}$$

In Equation (8), τ_RBD_ represents the estimated rigid body dynamics of the robot, as described in Equation (2). The BRNN is trained to approximate the ξ_ESID_ term, which is equivalent to the ξ term in Equation (1) and encompasses all unmodeled dynamics not captured by Equation (2). The difference between the torque commanded and the torque estimated by the τ_RBD_ model is used as the ground truth for ξ_ESID_.

**Figure 3:**
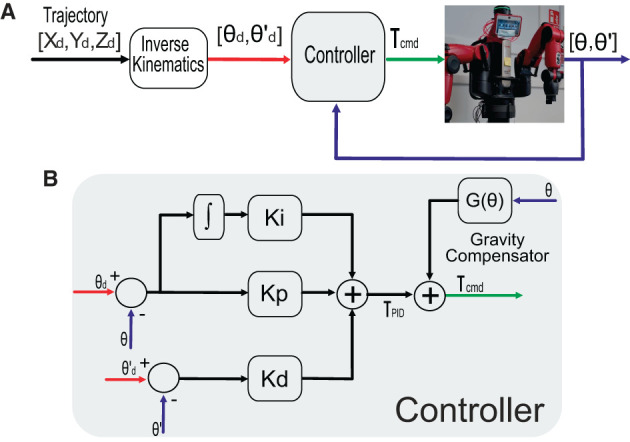
Schematic of the control system to obtain the dataset. **(A)** The general control system and **(B)** detailed PID controller plus gravity compensator.

The BRNN was implemented in Python using the Keras module running on TensorFlow. BRNN consisted of 64 hidden units that were trained with 32 batch sizes during 200 epochs. Adam Solver (Kingma and Ba, 2014) configured with a learning rate of 0.001 did optimize the loss function. The mean square error (MSE) metric was used as loss function for the actual and predicted torque values. The MSE metric is suitable for quantifying the difference between two sets of values when a small range of values is considered. Conversely, the mean absolute error (MAE) metric was used to compare the torque value predictions made by each proposed configuration at each joint. The MAE metric computes the average of the absolute difference between the actual and the predicted torque values (the actual values being those obtained from the dataset control loop, see Sections 2.4, 2.5 and Figure 3). The MAE and MSE metrics are described by


(9) $$\begin{array}{l} \text{MSE}=\frac{1}{N}\overset{N}{\underset{i=1}{\sum }}{\left(\tau_{i}-{\hat{\tau }}_{i}\right)}^{2}\text{ and} \end{array}$$

(10) $$\begin{array}{l} \text{MAE}=\frac{1}{N}\overset{N}{\underset{i=1}{\sum }}|\tau_{i}-{\hat{\tau }}_{i}|. \end{array}$$

In which, τ_*i*_ and ${\hat{\tau }}_{i}$ are the actual torque value and predicted torque value, respectively. *N* is the total number of data.

We also used the coefficient of determination, *r*^2^, which is defined as


(11) $$\begin{array}{l} r^{2}=1-\frac{\overset{N}{\underset{i=1}{\sum }}{\left(\tau_{i}-{\hat{\tau }}_{i}\right)}^{2}}{\overset{N}{\underset{i=1}{\sum }}{\left(\tau_{i}-\bar{\tau }\right)}^{2}} \end{array}$$

where


(12) $$\begin{array}{l} \bar{\tau }=\frac{1}{N}\overset{N}{\underset{i=1}{\sum }}\tau_{i}. \end{array}$$

*r*^2^ is a single value ranging from 0 to 1. This metric was used to evaluate and compare the overall performance of the three proposed configurations in predicting torque values for the seven joints.

### 2.4. Building the dataset: the torque control loop

Our BRNN demanded related torque vs. position/velocity data to be trained/tested. To that aim, we implemented a Baxter torque control scheme that allowed obtaining the joint commanded torque values. The torque-sensed values (τ_*s*_) represent the measured torque values in the joints, while the torque-commanded values (τ_*c*_) represent the desired torque values that the motors should exert on the joints. In the absence of collisions, τ_*s*_ and τ_*c*_ are correlated, but not identical. Since the robot's internal controller affects the actual torque applied to the joint, a dynamic behavior occurs between the commanded torque and the actual torque applied to the joint. This dynamic behavior is even more significant in cobots with a series of actuators (SEAs). Incorporating τ_*c*_ into the dynamic cobot model allows us to account for the dynamics of the cobot's internal controller, resulting in a more accurate representation of the cobot's behavior. In contrast, many data-based studies derive the dynamic cobot model from τ_*s*_ when they lack access to τ_*c*_ or only have position control, which may not capture the full dynamic behavior of the cobot. Torque-commanded values vs. robot state data provided more comprehensive robot information than the torque-sensed values used in other approaches (Liu et al., 2019). A more comprehensive robot dynamic dataset ultimately helps implement a more realistic inverse dynamic model.

The Robot Operating System (ROS) middleware was used to implement the torque control loop. A classical PID torque control plus gravity compensation was at the core of the control loop for trajectory tracking (Figure 3), thus obtaining actual positions, velocities, and commanded torque values per each desired trajectory (see Supplementary material). A 500-Hz sampling frequency was used for controlling, sensing, and data storing.

### 2.5. Building the dataset: trajectories tracked

The Baxter trajectory benchmark consisted of a set of random point-to-point movements for 1 min together with a set of closed periodic trajectories described by

(13) $$\begin{array}{l} x=r\;\text{sin}\left(\frac{2\pi }{T}t\right)+0.56,\\ y=r\;\text{cos}\left(\frac{2\pi }{T}t\right)+0.06,and\\ z=-0.22\;\text{cos}\left(\frac{2\pi }{T_{z}}t\right)+0.18,\\ \text{for }0\;\text{s}\leq t\leq 60\;\text{s} \end{array}$$

and

(14) $$\begin{array}{l} x=\left(r-\frac{0.01}{T_{z}}t\right)\text{sin}\left(\frac{2\pi }{T}t\right)+0.56,\\ y=\left(r-\frac{0.01}{T_{z}}t\right)\text{cos}\left(\frac{2\pi }{T}t\right)+0.06,and\\ z=-0.22\;\text{cos}\left(\frac{2\pi }{T_{z}}t\right)+0.18,\\ \text{for }0\;\text{s}\leq t\leq 8T_{z}\;\text{s}, \end{array}$$

instead of exclusively using a single periodic trajectory (Liu et al., 2019; Wang et al., 2020). These trajectories were selected to provide the greatest variety of information to the neural network (Kappler et al., 2017), i.e., covering as much workspace as possible at different speeds. To that aim, parameters *r*, *T*, and *T*_*z*_ varied accordingly (see Table 1). Each trajectory defined by (13) was executed for 1 min, whereas the three trajectories defined by (14) were executed during 80, 100, and 120 s, respectively, depending on the *T*_*z*_ value (see Table 1). The desired Cartesian space trajectories were converted to joint space using inverse kinematics (Coumans and Bai, 2016-2021); desired joint position and velocities were then fed to the PID controller, which generated the joint torque commands that resulted in actual joint positions and velocities (Figure 3). A continuous benchmark execution lasted 18 min (at a 500-Hz sampling frequency), which provided 540,000 data samples. Each data sample comprised position, velocity, and torque values per each Baxter joint. Note that the joint collisions, positions, and velocities constraints were accounted for.

**Table 1:** Trajectories tracked.

**Trajectory**	***T* (s)**	***r* (m)**	***T*_*z*_ (s)**
			15.0
		0.18	12.5
	2.5		10.0
			15.0
		0.15	12.5
Helical (13)			10
			12.0
		0.10	10.0
	2.0		8.0
			12.0
		0.05	10.0
			8.0
			10.0
Spiral (14)	2.5	0.18	12.5
			15.0

*T* is the period on the *x* and *y* axes; 2*r* is the motion amplitude in the *xy* plane; *T*_*z*_ is the period on the *z* axis.

Joint positions, velocities, and torque-commanded values were sampled at a frequency of 500 Hz and then stored for analysis. To create the learning sets, a cross-validation technique known as shuffle-split was used. This involved partitioning the total dataset into sub-sequences and shuffling them to create a training set (80% of samples) and a validation set (20% of samples). Normalization was performed on both the training and validation sets using the mean and standard deviation computed from the training set.

To facilitate the result replication and comparisons without needing to access the cobot, we made the dataset available at (see Supplementary material) https://github.com/EduardoRosLab/Baxter_Dynamic_Model.git.

### 2.6. Analyzing the GRU stability

Evaluating the stability of a system is crucial in any control problem. Input-State Stability (ISS) property of a GRU network (i.e., bounded inputs result in bounded NN states regardless of the initial condition; Limon et al., 2009) can be evaluated using a mathematical method proposed by Bonassi et al. (2021). For a GRU network to comply with ISS, the following condition must be satisfied:


(15) $$\begin{array}{c} v_{sb}<1, \end{array}$$

where


(16) $$\begin{array}{c} v_{sb}:=\frac{1}{4}\left(||U_{r}|{|}_{\infty }\left(||U_{\tilde{h}}|{|}_{\infty }+{\tilde{\sigma }}_{\tilde{h}}\right)+\frac{1+{\tilde{\phi }}_{r}}{1-{\tilde{\sigma }}_{z}}||U_{z}|{|}_{\infty }\right), \end{array}$$

and


$$\begin{array}{c} {\tilde{\sigma }}_{\tilde{h}}=\sigma \left(||W_{\tilde{h}}\;U_{\tilde{h}}\;b_{\tilde{h}}|{|}_{\infty }\right),\\ {\tilde{\sigma }}_{z}=\sigma \left(||W_{z}\;U_{z}\;b_{z}|{|}_{\infty }\right),and\\ {\tilde{\phi }}_{r}=tanh\left(||W_{r}\;U_{r}\;b_{r}|{|}_{\infty }\right). \end{array}$$

We apply (15) as a constraint during the training procedure to make our BRNN with GRU cells comply with the ISS property. The learning stability imposition to our BRNN shall decrease the torque command precision but ensure learning stability during the BRNN learning descent curve (Bonassi et al., 2021). By imposing learning stability during the BRNN learning descent curve, the analysis shall result in more robust weight values that could mitigate online learning problems, such as improper updating of network weights or/and instability in the learning process, that could negatively impact the performance of the BRNN and cause damage to the robot.

## 3. Results

### 3.1. Optimal temporal window and network size for the BRNN

The BRNN required selecting the number of forward and backward steps used to make the torque value predictions, i.e., temporal window size. To find the optimal trade-off between computational cost (time-window size) and BRNN resolution, we run the NID configuration with different time periods ranging from 6 to 70 ms. We performed the learning three times per time-window size. Cross-validation (shuffle-split) was used to create each learning set: 80% of the samples for the training set and 20% of samples for the validation set.

**Figure 4:**
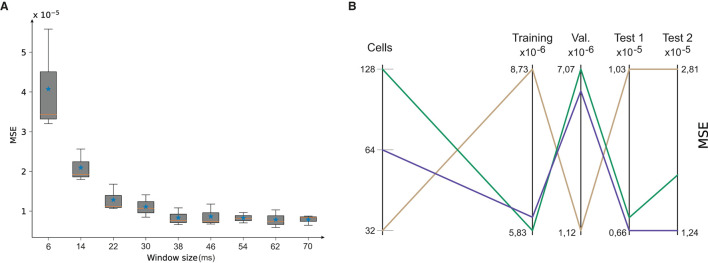
**(A)** Mean Squared Error (MSE) evolution after the NID configuration underwent training using different BRNN time-window sizes. **(B)** Network dimensionality analysis; the number of GRUs within the BRNN. The MSE indicates that for a number of 64 GRUs, our BRNN reaches lesser MSE values during the test phase; Test 1 was a circle trajectory whereas Test 2 was a squared trajectory. This dimensionality analysis indicates that a higher number of GRUs (128) leads to a lack of generalization, while a lower number (32) leads to a lack of precision in learning.

The results (Figure 4) depicted a precision (MSE) vs. time-window length (ms) exponential behavior. We found that the MSE average and variance did not substantially differ from time-window sizes larger than 46 ms. Accordingly, we used a 50-ms time-window size to maintain MSE values low at a minimum computational cost.

To determine the optimal GRU number of the BRNN, we explored values of 32, 64, and 128 cells. The MSE values achieved in training, validation, and prediction for non-trained circular (Test 1) and a square (Test 2) trajectory were taken into account (see Figure 4). According to these results a dimension of 64 GRU units was selected, as it provides a higher generalization of the data.

### 3.2. Trajectory benchmark analysis

**Figure 5:**
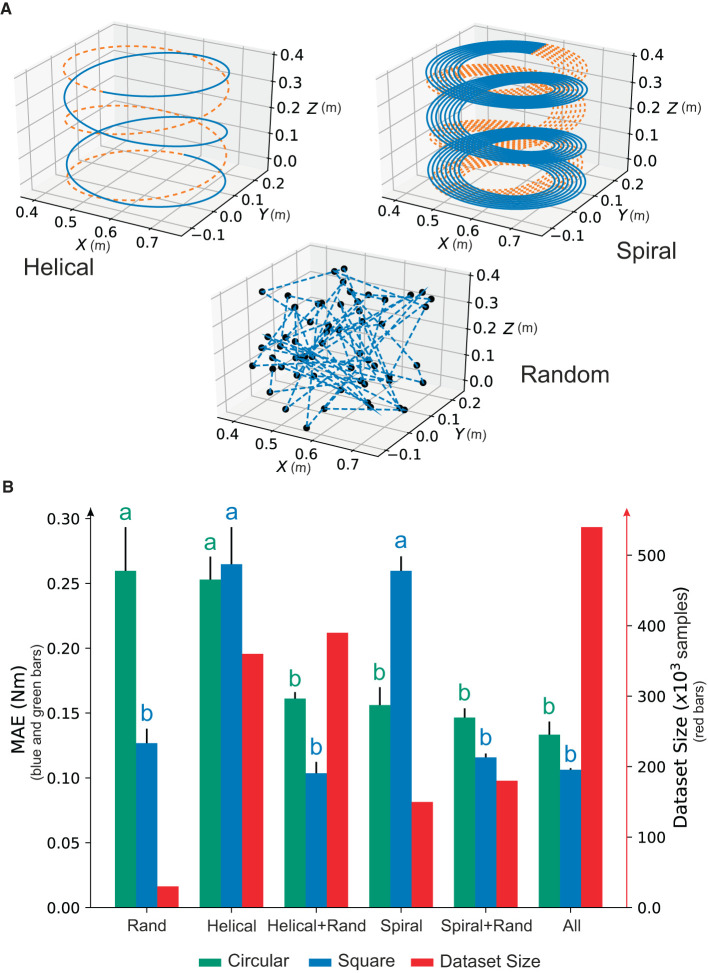
Selecting the training data subset. **(A)** Illustration of the trajectory set to be used **(B)**. A trade-off between precision and data subset size using the NID configuration following either circular or square non-trained trajectories. A different letter represents statistically significant differences found (ρ < 0.05) among the performance of the trajectory data subsets.

We trained the NID configuration using five different data subsets of trajectories defined within the benchmark (see Table 1) to quantify their capability of learning the inverse dynamic model from a minimum amount of data. We independently trained NID with (i) random trajectories only, (ii) helical trajectories only, (iii) helical and random trajectories, (iv) spiral trajectories only, and (v) spiral and random trajectories (Figure 5A).

We then used a non-trained circular and a square trajectory to test performance after training with each different subsets (i–v), and we also ran a one-way ANOVA with *post-hoc* Tukey analysis to compare the obtained results, i.e., the amount of information that each trajectory data subset brings to the inverse dynamic model.

Interestingly, we found non-significant statistical differences when performing the circular test trajectory for subsets (iii), (iv), and (v) compared to the full dataset. However, when performing the square test trajectory, whose shape substantially differed from any trajectory used during training, only subsets (iii) and (v) carried non-significant statistical differences compared to the full dataset (Figure 5B). We found that the random trajectories were instrumental in providing NID with generalization abilities for obtaining a precise inverse dynamic model, i.e., MAE significantly decreased (Figure 5B). The trajectory data subset (v) was chosen as the training dataset since it matched performance with the full dataset but using a lesser amount of data (180,000 vs. 540,000; see red bars in Figure 5B).

### 3.3. Precision of NID, SID, and ESID configurations

**Figure 6:**
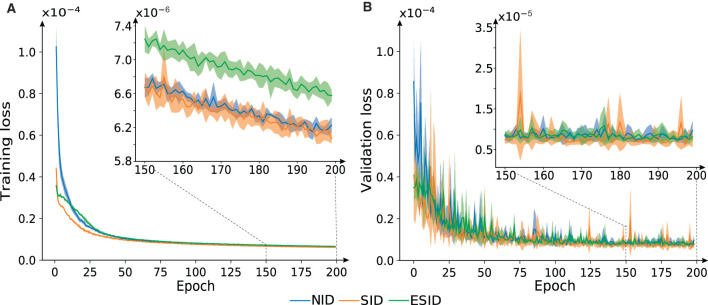
Evolution of the loss function for NID, SID, and ESID configurations. **(A)** Training and **(B)** test datasets. **(A, B)** Zoom-in depicts how overfitting is prevented.

Each configuration, namely NID, SID, and ESID (as described in Section 2), underwent independent training for three runs. Cross-validation was employed to generate training/validation sets for each run. The data set used for this purpose was obtained from the trajectory subset (v) mentioned earlier (Figure 6). We found that, in terms of error accuracy, these three configurations behaved similarly during the training phase. SID and ESID started the training from lower MSE values than NID due to the inverse RBD model information provided to the BRNN. However, they all converged to the same MSE value in 50 iterations (Figure 6). We stopped learning after 200 epochs to prevent the three configurations from overfitting the training dataset, thus preserving the configuration capacity for generalization.

**Figure 7:**
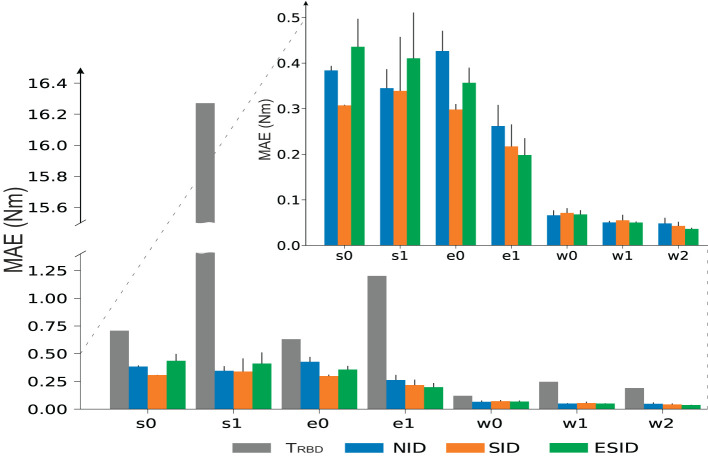
Comparison among the NID, SID, and ESID proposed configurations and the analytical rigid body dynamics model.

We then used the MAE metric to compare the performance per cobot joint of the aforementioned configurations, i.e., NID (see Supplementary Figure 1), SID, and ESID, compared to the performance of the rigid body dynamic model of the cobot described (2). We found that any of the three configurations (Figure 7) largely outperformed the manufacturer RBD model (MAE values: NID 0.15 ± 0.02 Nm, SID 0.12 ± 0.02 Nm, and ESID 0.12 ± 0.02 Nm, RBD 3.83 Nm). The performance improvement was particularly remarkable at the S1 joint (shoulder). Baxter holds an external spring (elastic passive component) that facilitates the shoulder to fix position (Fitzgerald, 2013). This external spring is not even considered within the RBD model, which would explain much of this significant difference.

Importantly, we found no statistical difference among the three configurations (NID, SID, and ESID) when comparing performance (MAE) by ANOVA tests. We must, however, also highlight that the NID configuration did not use prior information provided by the RBD model, which is often not available (Smith and Mistry, 2020; Huang et al., 2021).

For comparative purposes, our NID configuration was also implemented using LSTM cell units. We found similar validation errors for LSTM and GRU cell implementations (i.e., MSE = 6.40*10^−6^ vs. MSE = 6.27*10^−6^, respectively), but a faster processing speed using the GRU cell implementation (i.e., 5,600 sample set computation time takes 640 ms LSTM vs. 230 ms GRU). In light of these results, and being RT computing a limiting factor in robotics, we finally chose to use the GRU cell implementation. To compare the performance of our BRNN architecture, we also tested two other architectures: a multilayer perceptron (MLP) with three hidden layers and 64-128-64 neurons and a unidirectional RNN with 64 GRU cells. Both RNNs performed better than MLP. This suggests that using information from both past and future time steps is important for learning the inverse cobot dynamics. The results are summarized in Table 2.

**Table 2:** Inverse dynamic model torque prediction over different test trajectories.

	**MAE**						***r*** ^**2**^		
**Path**	**RBD**	**MLP**	**RNN**	**NID**	**SID**	**ESID**	**NID**	**SID**	**ESID**
Circular XY	3.83	1.73	0.48	**0.15**	**0.15**	**0.15**	**0.92**	**0.92**	**0.92**
Helical	3.27	1.62	0.43	0.22	**0.19**	0.22	0.89	0.87	**0.90**
Circular XZ	3.17	1.91	0.32	0.17	**0.15**	0.16	**0.82**	0.79	0.80
Square	4.37	2.25	0.51	0.27	**0.21**	0.23	0.72	**0.76**	**0.76**

The bold values indicate the best results.

### 3.4. Evaluating NID, SID, and ESID configurations: generalization of the inverse dynamic model

To assess the generalization capabilities of the NID, SID, and ESID configurations, their inverse dynamics models faced a series of trajectories (test set) neither used during training nor validation: (i) circular trajectories on the XY and XZ plane, (ii) a helical trajectory, and (iii) a square trajectory (see Supplementary material). An inverse dynamic model, which can generalize, shall predict precise torque values for any trajectory regardless the training/validation trajectory used.

To analyze and quantify how good each inverse dynamic model was at learning from the given dataset and applying the learned information to new trajectories neither used during training nor validation, we obtained their MAE values per joint and their *r*^2^ values. MAE allowed us to compare individual joint precision (see Supplementary material), whereas *r*^2^ provided for the correlation between the predicted and actual torque values in a single value measurement.

We found that the generalization ability for the NID, SID, and ESID configurations remained similar. Importantly, the more alike the new non-trained trajectories (test set) were to the trained trajectories, the higher *r*^2^ is obtained (0.92 and 0.90, see Table 2) and vice versa (0.82 and 0.76, see Table 2). Despite NID, SID, and ESID comparable performance, no prior knowledge of analytical dynamics for NID configuration is needed.

### 3.5. Case study: real-time, real-world, feed-forward control

**Figure 8:**
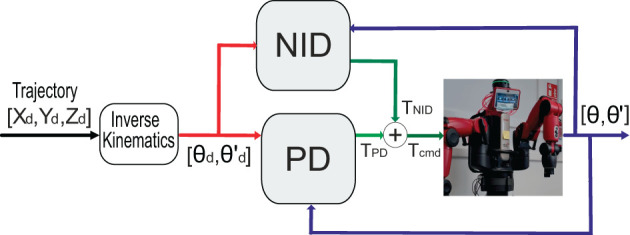
NID implemented as a feedforward controller together with a PD feedback controller.

Using the inverse dynamic robot model as a controller is of common use (Jordan and Rumelhart, 1992; Stogiannos et al., 2018). Here, we propose our NID configuration as a feedforward controller working conjointly with a feedback PD controller (Figure 8). We developed this case study under the assumption that a better dynamic cobot model shall require less action from the feedback control loop (PD). With this implementation, we could indirectly verify how closely the learned inverse dynamic model matched the real robot. It is important to note that this case study's main aim was not outperforming a PD control but to demonstrate one of the many potential applications of the inverse dynamic model of a collaborative robot. NID configuration is based on a worst-case control scenario compared to SID and ESID since the RBD model is unknown and thus the entire robot dynamics shall be learned. The NID configuration represents not just a theoretical RNN implementation but rather it aims at a robotic real-world RNN control application. Once we trained our NID configuration, we used it as a real feedforward controller (Figure 8). NID estimated the torque values to be provided per joint to follow a desired trajectory (circular trajectory used as reference). The input data to the NID were actual and desired robot states (positions and velocities) of the robot joints.

**Figure 9:**
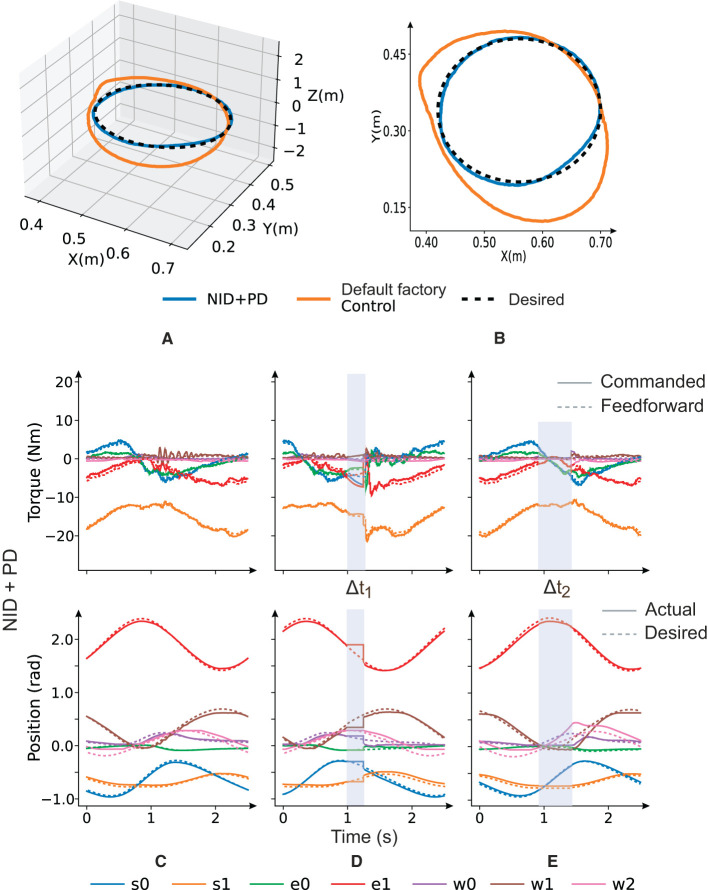
Testing the NID + PD control performance using a circular trajectory as reference. Default factory control vs. feedforward NID + PD torque control **(A, B)**. Testing torque value estimation accuracy and PD control contribution are negligible **(C)**. Testing compliance and response to disturbances **(D)**, NID + PD torque response during a 250-ms total locking of all robot joints Δ*t*_1_ = 250 ms. Testing NID resilience **(E)**, feedforward control temporally without feedback control during Δ*t*_2_ = 500 ms.

Four scenarios were proposed to test the capability of our control system to track a circular trajectory as reference:

1. *Stability analysis:* Our NID configuration was trained excluding/including the ISS constraint (15) within the loss function as described by Bonassi et al. (2021). Predictably, the NID accuracy performance decreased when imposing the ISS stability criteria (see Table 3, NID vs. NIDsb). In this case study example, we prioritized NID accuracy and accompanied the NID controller in a feedforward loop with a PD in a feedback loop to compensate for small NID torque value estimation errors and to ensure closed-loop system stability (Hu et al., 2021). Note that the stability depends on the PD closed-loop characteristics rather than on the feedforward GRU term that only provides for precomputed torque values.
2. *Torque estimation accuracy:* We performed a circular trajectory of radius 0.15 m in the XY plane using our proposed controller (NID + PD), as shown in Figures 9A, B. The resulting mean absolute error (MAE) of the end-effector position for NID + PD (7.0*10^−^3 m) was significantly lower than the MAE of the end-effector position achieved by the robot's default factory position controller (79.0*10 − 3 m; see Table 4). Our analysis revealed that the NID controller executed the majority of the control action, while the contribution of the PD controller remained residual (see Figure 9C). This suggests that the torque estimated by the NID was closely aligned with the torque required to track the reference trajectory (see Supplementary material).
3. *Compliance and response to disturbances:* We programmed a total locking of all joints during 250 ms at time Δ*t*_1_ (Figure 9D). During the locking period, the NID + PD control did not execute high torque values to compensate for the error, as a PD controller without the feedforward component would do. Thus, compliance was improved due to the lower energy at stake (Figure 9D). We also found the NID + PD control to better deal with the disturbances. After the locking period, NID + PD control almost instantaneously resumed tracking the reference trajectory without a significant transient converging stage (Figure 9D; see Supplementary material).
4. *NID Resilience in feedforward control:* We also tested the capability of the NID to track the trajectory in open-loop torque control. To do so, we switched off the PD feedback control action (τ_*PD*_ = 0) during 500 ms (Δ*t*_2_) and confirmed that NID could seamlessly track the trajectory (see Figure 9E) in the absence of active feedback control (see Supplementary Figure 2).

**Table 3:** Comparison of performance between NID trained with and without considering the ISS property.

	***v*_*sb*_ [GRU_*b*_, GRU_*f*_] (16)**	**ISS condition (15)**	**MSE training**	**MSE validation**
NID	[inf, inf]	No	6.17*10^−6^	6.27*10^−6^
NID_*sd*_	[0.15, 0.17]	Yes	1.83*10^−5^	2.26*10^−5^

**Table 4:** Performance on a circular trajectory of the default factory controller and the NID + PD controller.

		**Default factory**	**NID + PD**
	s0	0.112	**0.033**
	s1	0.034	**0.005**
Joint MAE (rad)	e0	0.027	**0.007**
		e1	0.151	0. **024**
	w0	**0.014**	0.021
	w1	0.042	**0.035**
	w2	**0.023**	0.034
End-effector MAE (m)		0.079	**0.007**

The bold values indicate the best results.

## 4. Discussion

The presented ML approach identified the inverse dynamic model of a cobot (Baxter) using a BRNN with GRUs at the core. These BRNNs, in turn, were adapted and integrated into NID, SID, and ESID configurations. It is noteworthy that, the GRUs eliminated the vanishing gradient problem in the temporal input values by keeping the relevant temporal information and passing it down to the next time steps of the BRNN network (Yang et al., 2020). We found that the larger the sliding time window, which was partially containing and feeding the GRUs with temporal input values, the better the performance of Baxter's inverse dynamic model. However, the computational load also increased with the size of the time window, which ultimately jeopardized Baxter's Real Time (RT) operation. The temporal window size trade-off found at 50 ms ensured Baxter's RT operation with no significant loss of accuracy with respect to longer time windows (Francis, 1995). This is remarkable, in contrast to other studies with large temporal windows (Liu et al., 2019; Wang et al., 2020) accounting for precision only and sidestepping RT robot operation. The proposed BRNN algorithms are designed to identify the inverse dynamics of a robot, treating the robot as a time-invariant system. To account for real-time performance effects, such as backlash or non-linearities from friction or elastic components, offline learning of the inverse dynamics is performed using data recorded during the execution of trajectories on a real robot. This incorporation of real-time effects leads to more accurate learned dynamics. Selecting the BRNN sliding time window length was essential, but also the data contained in it. We considered the question of selecting those representative trajectory data that would contribute most effectively to the BRNN's generalization capabilities.

Needless to say, there are endless possibilities to explore Baxter's workspace, hence selecting those trajectories that better reveal its non-linear dynamics was pivotal. We found that combining random trajectories together with cyclic ones (modulating their magnitude and frequency, see Section 2) while exploring Baxter's workspace revealed better its non-linear dynamics rather than using cyclic or random exploration only, i.e., data diversity vs. data quantity. The use of this representative training/validation dataset endowed the NID, SID, and ESID configurations with similar generalization capabilities when predicting torque values from their corresponding Baxter's inverse dynamic models. As assumed by Kappler et al. (2017), we verified the superior performance of those configurations using rigid-bodied analytic dynamic modeling supporting BRNN (SID and ESID) when predicting torque values under dissimilar trajectories to the original training set. However, the differences found were not statistically significant among the three configurations during validation. Importantly, NID, unlike SID and ESID configurations, operated with no prior knowledge of Baxter's dynamics, which makes the NID configuration more suitable for robotic applications in which the analytic dynamic model of the robotic agent is either mathematically intractable or not available, i.e., soft robots and elastic robots. Conversely, SID and ESID could benefit from employing the analytical dynamic model when a cobot faces an unexplored working space. Finally, we tested NID configuration as a proof of concept in a real-time, real-control scenario, i.e., NID as a feedforward controller together with a PD controller that helped compensate for small torque estimation errors. Interestingly, model-free control approaches can also be used to control robots with complex modeling dynamics (Bian et al., 2020). These approaches can provide a controller with excellent performance for a specific task, even without an accurate model of the robot (Tutsoy et al., 2023). However, in these methods, the controller and the dynamic model usually operate as a whole, which might limit the independent use of the obtained dynamic model.

The NID configuration, as demonstrated in Sections 3.3 and 3.4, has been shown to have high torque estimation performance. This makes it a good candidate for real-time control applications where the inverse dynamics of the robot are the main actions, as implemented in Section 3.5. In real-time scenarios, there is a risk of joint locking, collisions, errors, or delays in sensing the robot's state. However, the proposed implementation deals with these situations by maintaining the real robot close to the desired trajectory with compliant features, as described in Section 3.5. It should be noted, though, that caution is required when working with trajectories outside the workspace of the training-validation dataset. Since NID is a non-parametric algorithm, its performance in data extrapolation cannot be guaranteed (Mozian et al., 2020). If the real robot is going to work outside the explored space, it would be more advisable to use a semi-parametric inverse dynamic configuration, such as those presented in Section 2.5. We confirmed that the NID + PD controller outperformed the robot's default factory position controller in terms of path tracking accuracy. The NID also helped improve the PD controller in terms of (i) compliance and response to disturbances and (ii) resilience to open-loop control at short time intervals. Note that, as aforementioned, we implemented NID as a worst-case scenario feedforward robot controller. Given a RBD robot model, SID and ESID could also be implemented. Improved torque estimation is one of the main control goals when operating cobots in unstructured scenarios involving human-robot interaction (HRI). Not only the energy at stake is diminished preventing damage in case of collision but also the improved precision and response to disturbances help avoid those collisions (Mohajerin and Waslander, 2019). We also verified the NID resilience by depriving temporally the control architecture of any active feedback information during 500 ms. NID controller was able to keep track of the reference trajectory with precision. The NID capacity of operating with no feedback could be useful in remote control, cloud, or fog computing scenarios, presenting significant non-deterministic latencies in the transference of sensed information. NID could indeed predict the commanded torque values while temporally blocking the sensorimotor feedback, i.e., during open loop operation.

It is important to note that the mechanical and electrical components of a robot may undergo changes in their parameters over time due to factors, such as environmental conditions or wear, resulting in dynamic changes in the cobot (Camoriano et al., 2016). Significant differences between the torque estimated by the learned inverse dynamic model and the torque sensed at the joints may be indicative of such changes in the parameters of the real robot. In such cases, the learned offline dynamic cobot model would no longer be effective for controlling the cobot, and retraining would be necessary after cobot maintenance or repair. There are online learning strategies available for training a BRNN in real-time applications, if needed. However, training the Bidirectional Recurrent Neural Network (BRNN) in real time can be challenging due to multiple factors. These factors include the risk of fatal errors that may occur if the network weights are updated improperly, as well as the potential for a delayed control loop if the computational cost of training the model on real-time data is significant. Furthermore, if the goal is to identify sudden changes in the robot's dynamics caused by external factors, it may be more appropriate to leave the dynamic model invariant during the task execution to prevent any potential issues. Since data-driven approaches have no analytical guarantee of performance, they may be difficult to certify for safety critical applications. This can be mitigated by integrating a PD controller into the loop to account for unexpected uncertainties. Furthermore, if deviations from the expected performance are encountered (for instance detectable by measuring the error or the PD output activity), safety actions, such as interlock or alarm signals, shall be integrated. In future studies, the performance of the PD controller used in the feedforward loop in this study could be compared with other adaptive control strategies for cobots, such as the ones proposed by Pan et al. (2018). Additionally, the possibility of integrating our NID configuration with more robust control architectures or adaptive control strategies could be explored to assess how the balance between identification techniques and control techniques would be.

## Data availability statement

The original contributions presented in the study are included in the article/Supplementary material, further inquiries can be directed to the corresponding author.

## Author contributions

BV-V and NL conceived the article's initial idea, designed, and modeled and implemented the set-up experimentation. They also prepared the figures, drafted the manuscript, reviewed the manuscript, and approved the final version. IA drafted the manuscript, reviewed the manuscript, and approved the final version. ER conceived the article's initial idea, drafted the manuscript, reviewed the manuscript, and approved the final version.
