# Supplementary material for: Bidirectional recurrent learning of inverse dynamic models for robots with elastic joints: a real-time real-world implementation
Source: Front Neurorobot. 2023 Jun 16;17:1166911. doi: 10.3389/fnbot.2023.1166911 (PMC10311998; doi:10.3389/fnbot.2023.1166911)
Supplement: Supplementary file 3 [file Data_Sheet_2.pdf]

frontiers

Supplementary Material

1 OBTAINING THE DATA SET: THE PID GAINS

We first used the robot simulator to find those PID control gains that optimized the integral of absolute error (IAE) performance function (equation S1). Then, starting from these previous values, we further adjusted these gains for each joint using the actual Baxter robot. The gains used to generate the trajectories are listed in the Table S1.

$$IAE = \int ||e(t)||dt \tag{S1}$$

Table S1. PID Control Gains

Joint	Kp	Ki	Kd
S0	50.0	2.0	8.5
S1	80.0	0.8	11.0
E0	80.0	0.6	1.6
E1	120.0	4.5	10.0
W0	80.0	0.6	1.6
W1	40.0	0.6	1.6
W2	40.0	0.8	1.6

2 THE DATASET

URL containing the training and testing data that were used for the BRNN to learn the inverse dynamics of the Baxter robot. The dataset consisted of temporal sequences of join angles and velocities corresponding to a sequence of applied torque values tracking the benchmark trajectories proposed: `https://github.com/EduardoRosLab/Baxter_Dynamic_Model.git`.

3 TORQUE PREDICTED BY NID CONFIGURATION

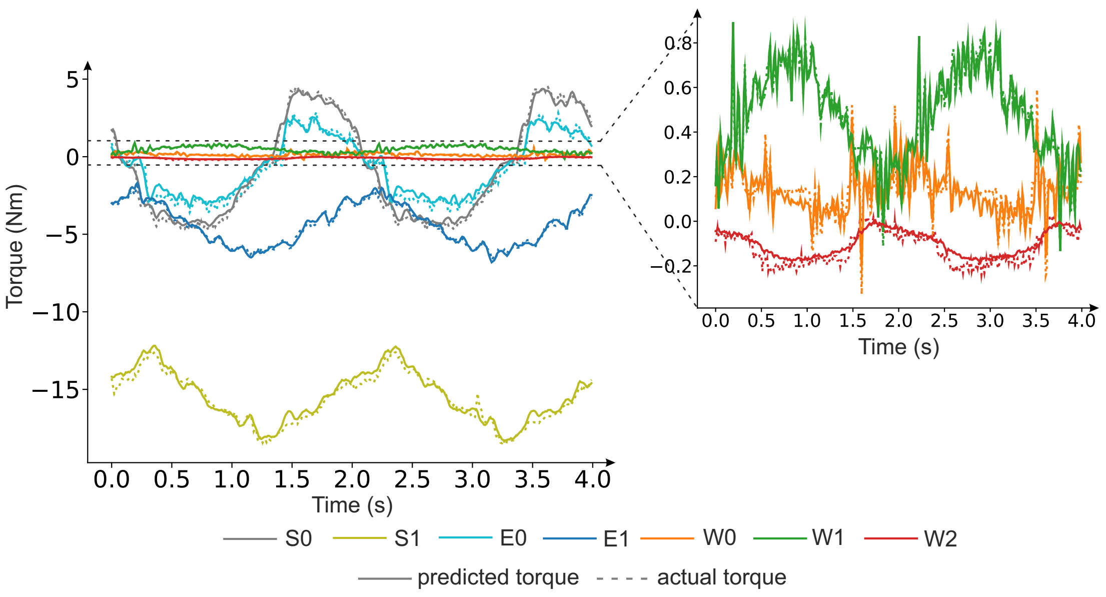

Figure S1. Torque values per Baxter's joint estimated by the NID configuration following a circular trajectory as reference. Zoom in shows those torque joint values with lesser range.

4 TEST SET

Four trajectories were used as test set to evaluate the performance of the trained models.

- Circular trajectory on the XY plane:

$$x = 0.15 \sin\left(\frac{2\pi}{2.5}t\right) + 0.56,$$
$$y = 0.15 \cos\left(\frac{2\pi}{2.5}t\right) + 0.33,$$
$$z = 0.10. \tag{S2}$$

- Circular trajectory on the XZ plane:

$$x = 0.10 \sin\left(\frac{2\pi}{2.5}t\right) + 0.56,$$
$$y = 0.06,$$
$$z = 0.10 \cos\left(\frac{2\pi}{2.5}t\right) + 0.18. \tag{S3}$$

- Helical trajectory:

$$x = 0.18 \sin\left(\frac{2\pi}{2.5}t\right) + 0.56,$$
$$y = 0.18 \cos\left(\frac{2\pi}{2.5}t\right) + 0.06,$$
$$z = -0.22 \cos\left(\frac{2\pi}{12.5}t\right) + 0.18. \tag{S4}$$

- Square trajectory with vertices:

$$V_1 = (0.46, -0.06, 0.05),$$
$$V_2 = (0.76, -0.06, 0.05),$$
$$V_3 = (0.76, 0.24, 0.05),$$
$$V_4 = (0.46, 0.24, 0.05). \tag{S5}$$

5 INVERSE DYNAMIC MODEL TORQUE PREDICTION OVER DIFFERENT TEST TRAJECTORIES.

Table S2. MAE of the Torque Prediction per Joint over Different Test Trajectories.

	Circular Path XY				Helical Path			
Joint	RBD	NID	SID	ESID	RBD	NID	SID	ESID
S0	0.94	0.27 ± 0.02	0.22 ± 0.01	0.23 ± 0.01	0.88	0.42 ± 0.01	0.32 ± 0.01	0.49 ± 0.04
S1	23.61	0.29 ± 0.03	0.3 ± 0.02	0.29 ± 0.07	19.57	0.4 ± 0.04	0.35 ± 0.01	0.37 ± 0.06
E0	0.84	0.22 ± 0.03	0.22 ± 0.02	0.23 ± 0.01	0.76	0.36 ± 0.03	0.29 ± 0.03	0.3 ± 0.01
E1	0.87	0.13 ± 0.01	0.16 ± 0.03	0.16 ± 0.03	1.08	0.21 ± 0.02	0.2 ± 0.02	0.19 ± 0.02
W0	0.15	0.06 ± 0.01	0.05 ± 0.01	0.05 ± 0.01	0.13	0.07 ± 0.01	0.07 ± 0.01	0.08 ± 0.01
W1	0.29	0.03 ± 0.00	0.04 ± 0.01	0.05 ± 0.01	0.26	0.05 ± 0	0.06 ± 0.01	0.06 ± 0.02
W2	0.12	0.03 ± 0.01	0.03 ± 0.01	0.03 ± 0.01	0.19	0.05 ± 0.02	0.05 ± 0.01	0.04 ± 0.01
	Circular Path XZ				Square Path			
Joint	RBD	NID	SID	ESID	RBD	NID	SID	ESID
S0	0.94	0.28 ± 0.03	0.26 ± 0.02	0.27 ± 0.02	1.04	0.36 ± 0.03	0.3 ± 0.01	0.37 ± 0.03
S1	18.98	0.33 ± 0.04	0.28 ± 0.05	0.32 ± 0.03	27.51	0.66 ± 0.05	0.43 ± 0.14	0.54 ± 0.11
E0	0.69	0.24 ± 0.04	0.2 ± 0.02	0.23 ± 0.00	0.84	0.4 ± 0.03	0.32 ± 0.02	0.25 ± 0.02
E1	1.08	0.17 ± 0.02	0.15 ± 0.03	0.17 ± 0.01	0.73	0.21 ± 0.01	0.17 ± 0.03	0.2 ± 0.02
W0	0.09	0.06 ± 0.01	0.05 ± 0.01	0.05 ± 0.00	0.16	0.09 ± 0.04	0.09 ± 0.03	0.09 ± 0.02
W1	0.29	0.04 ± 0.00	0.04 ± 0.00	0.05 ± 0.01	0.16	0.17 ± 0.01	0.13 ± 0.02	0.15 ± 0.04
W2	0.09	0.04 ± 0.00	0.06 ± 0.01	0.05 ± 0.00	0.14	0.04 ± 0.01	0.05 ± 0.01	0.04 ± 0.00

6 NID FEEDFORWARD CONTROL DEPLOYMENT ON THE REAL ROBOT

The video depicts the Baxter controlled by the NID neural network in a feedforward loop. We compared the NID performance (accuracy) to a conventional PD. We also compared their performances under perturbations. We finally showed NID ability (Fig. S2) to track the reference trajectory in the absence of active feedback control: `https://github.com/EduardoRosLab/Baxter_Dynamic_Model.git`.

7 BACKPROPAGATION THROUGH TIME CODE

Backpropagation through time (BPTT) is a gradient-based optimization algorithm for training recurrent neural networks (RNNs). BPTT applies the backpropagation algorithm to a unrolled version of the RNN, where the network is unfolded in time steps. This results in a feedforward network with multiple layers and shared weights across time steps. The algorithm computes the gradients of the loss function with respect to the weights and biases of the network by propagating the error backwards through time from the final time step to the first.

Input:

- $x_1, x_2, ..., x_T$: input sequence
- $y_1, y_2, ..., y_T$: target sequence
- θ: parameters of the recurrent neural network (RNN)

Output:

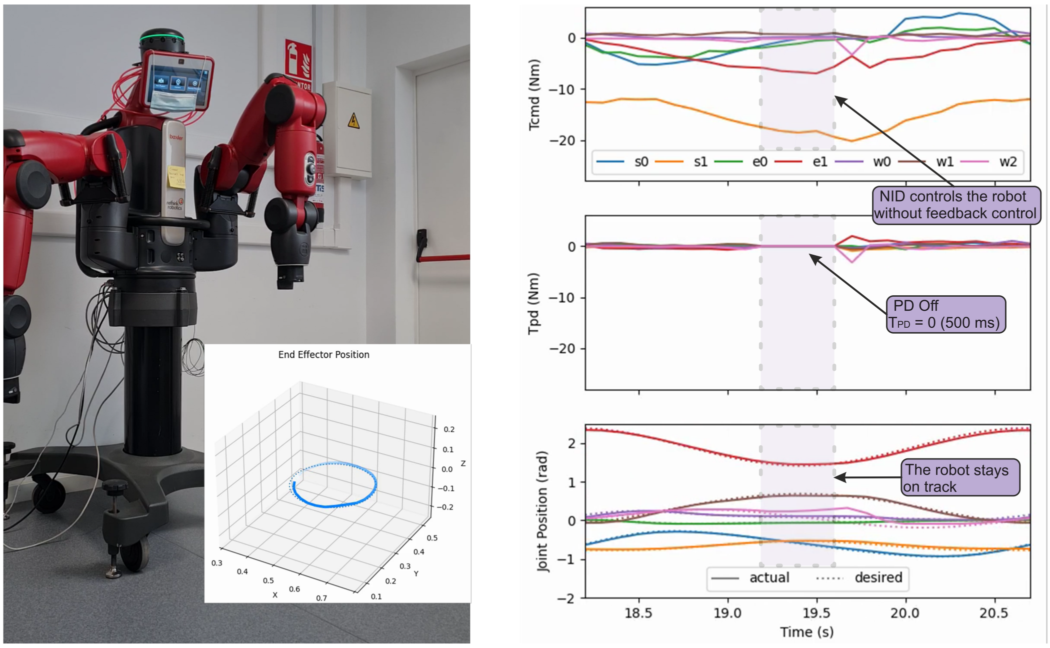

Figure S2. Performance of NID implemented on the real robot (Video capture).

- $\nabla_\theta \mathcal{L}$: gradient of the loss with respect to the parameters

1. Initialize the hidden state h_0 of the RNN to zero
2. Forward pass - For each time step t in the input sequence:
   - Compute the output o_t and the hidden state h_t using the current input x_t and the previous hidden state h_{t-1}: $o_t, h_t = RNN(x_t, h_{t-1}; \theta)$
   - Compute the loss $\mathcal{L}_t$ between the target y_t and the output o_t: $\mathcal{L}_t = \mathcal{L}(y_t, o_t)$
3. Initialize the gradient of the loss with respect to the parameters: $\nabla_\theta \mathcal{L} \leftarrow 0$
4. Backward pass - For each time step t in reverse order:
   - Compute the gradient of the loss with respect to the output at time step t: $\delta_t \leftarrow \nabla_{o_t} \mathcal{L}_t$
   - Compute the gradient of the loss with respect to the parameters and the hidden state at time step t: $\nabla_\theta \mathcal{L}t, \nabla h_t \mathcal{L}t \leftarrow$ Backprop$(x_t, ht-1, \theta, \delta_t)$
   - Accumulate the gradient of the loss with respect to the parameters: $\nabla_\theta \mathcal{L} \leftarrow \nabla_\theta \mathcal{L} + \nabla_\theta \mathcal{L}_t$
5. Return the gradient of the loss with respect to the parameters: $\nabla_\theta \mathcal{L}$

Note: the function Backprop$(x_t, h_{t-1}, \theta, \delta_t)$ computes the gradient of the loss with respect to the parameters and the hidden state at time step t using the chain rule and backpropagation. This function depends on the specific architecture of the RNN and the loss function used.
